# Supplementary material for: Resolving the full spectrum of human genome variation using Linked-Reads
Source: Genome Res. 2019 Apr;29(4):635–45. doi: 10.1101/gr.234443.118 (PMC6442396; doi:10.1101/gr.234443.118)
Supplement: Supplemental Material [file supp_gr.234443.118_Supplemental_longranger-2.2.zip › longranger-2.2.tar.bz2!/lr2.2-public/lib/stan/cmdstan/stan/doc/api/html/math_2matrix_2multiply_8hpp_source.html]

Stan: src/stan/math/matrix/multiply.hpp Source File


|  |  |
| --- | --- |
|  | Stan  2.5.0  probability, sampling & optimization |


- Main Page
- Related Pages
- Namespaces
- Classes
- Files

- File List
- File Members

All Classes Namespaces Files Functions Variables Typedefs Enumerations Enumerator Friends Macros Pages

- src
- stan
- math
- matrix

multiply.hpp

Go to the documentation of this file.

1 #ifndef STAN\_\_MATH\_\_MATRIX\_\_MULTIPLY\_HPP

2 #define STAN\_\_MATH\_\_MATRIX\_\_MULTIPLY\_HPP

3

4 #include <boost/type\_traits/is\_arithmetic.hpp>

5 #include <boost/utility/enable\_if.hpp>

6 #include <stan/math/matrix/Eigen.hpp>

7 #include <stan/math/error\_handling/matrix/check\_matching\_sizes.hpp>

8 #include <stan/math/error\_handling/matrix/check\_multiplicable.hpp>

9

10 namespace stan {

11  namespace math {

12

21  template <int R, int C, typename T>

22  inline

23  typename boost::enable\_if\_c<boost::is\_arithmetic<T>::value,

24  Eigen::Matrix<double, R, C> >::type

25  multiply(const Eigen::Matrix<double, R, C>& m,

26  T c) {

27  return c \* m;

28  }

29

30  // FIXME: apply above pattern everywhere below to remove

31  // extra defs, etc.

32

41  template <int R, int C, typename T>

42  inline

43  typename boost::enable\_if\_c<boost::is\_arithmetic<T>::value,

44  Eigen::Matrix<double, R, C> >::type

45  multiply(T c,

46  const Eigen::Matrix<double,R,C>& m) {

47  return c \* m;

48  }

49

60  template<int R1,int C1,int R2,int C2>

61  inline Eigen::Matrix<double,R1,C2> multiply(const Eigen::Matrix<double,R1,C1>& m1,

62  const Eigen::Matrix<double,R2,C2>& m2) {

63

64  stan::math::check\_multiplicable("multiply(%1%)",m1,"m1",

65  m2,"m2",(double\*)0);

66  return m1\*m2;

67  }

68

78  template<int C1,int R2>

79  inline double multiply(const Eigen::Matrix<double,1,C1>& rv,

80  const Eigen::Matrix<double,R2,1>& v) {

81  stan::math::check\_matching\_sizes("multiply(%1%)",rv,"rv",

82  v,"v",(double\*)0);

83  if (rv.size() != v.size())

84  throw std::domain\_error("rv.size() != v.size()");

85  return rv.dot(v);

86  }

87

88  }

89 }

90 #endif

check\_multiplicable.hpp

check\_matching\_sizes.hpp

stan::math::check\_multiplicable

bool check\_multiplicable(const char \*function, const T1 &y1, const char \*name1, const T2 &y2, const char \*name2, T\_result \*result)

**Definition:** check\_multiplicable.hpp:15

Eigen.hpp

stan::math::check\_matching\_sizes

bool check\_matching\_sizes(const char \*function, const T\_y1 &y1, const char \*name1, const T\_y2 &y2, const char \*name2, T\_result \*result)

**Definition:** check\_matching\_sizes.hpp:15

stan::math::multiply

boost::enable\_if\_c< boost::is\_arithmetic< T >::value, Eigen::Matrix< double, R, C > >::type multiply(const Eigen::Matrix< double, R, C > &m, T c)

Return specified matrix multiplied by specified scalar.

**Definition:** multiply.hpp:25


---

[ Stan Home Page ]

*© 2011–2014,
Stan Development Team.*
